# Stress Hyperglycemia, Insulin Treatment, and Innate Immune Cells

**DOI:** 10.1155/2014/486403

**Published:** 2014-05-08

**Authors:** Fangming Xiu, Mile Stanojcic, Li Diao, Marc G. Jeschke

**Affiliations:** ^1^Ross Tilley Burn Centre, Sunnybrook Health Sciences Centre, University of Toronto, 2075 Bayview Avenue, Room D704, Toronto, ON, Canada; ^2^Sunnybrook Research Institute, University of Toronto, Toronto, ON, Canada M4N 3M5; ^3^Department of Surgery, Division of Plastic Surgery, Department of Immunology, University of Toronto, Toronto, ON, Canada

## Abstract

Hyperglycemia (HG) and insulin resistance are the hallmarks of a profoundly altered metabolism in critical illness resulting from the release of cortisol, catecholamines, and cytokines, as well as glucagon and growth hormone. Recent studies have proposed a fundamental role of the immune system towards the development of insulin resistance in traumatic patients. A comprehensive review of published literatures on the effects of hyperglycemia and insulin on innate immunity in critical illness was conducted. This review explored the interaction between the innate immune system and trauma-induced hypermetabolism, while providing greater insight into unraveling the relationship between innate immune cells and hyperglycemia. Critical illness substantially disturbs glucose metabolism resulting in a state of hyperglycemia. Alterations in glucose and insulin regulation affect the immune function of cellular components comprising the innate immunity system. Innate immune system dysfunction via hyperglycemia is associated with a higher morbidity and mortality in critical illness. Along with others, we hypothesize that reduction in morbidity and mortality observed in patients receiving insulin treatment is partially due to its effect on the attenuation of the immune response. However, there still remains substantial controversy regarding moderate versus intensive insulin treatment. Future studies need to determine the integrated effects of HG and insulin on the regulation of innate immunity in order to provide more effective insulin treatment regimen for these patients.

## 1. Stress Hyperglycemia in Critical Illness

The effects of severe trauma, infection, and surgery result in remarkable metabolic stress on the human body. Stress associated with critical illness is characterized by the activation of inflammatory cellular mediators and the hypothalamic-pituitary-adrenal (HPA) axis. The release of cortisol, catecholamines, glucagon, and growth hormone is an essential component of the general adaptation to illness and stress. The acute response to critical illness (energy conservation toward vital organs, modulation of the immune system, and a delay in anabolism) is generally considered to be an appropriate and adaptive response that occurs in the first few days after insult. Mild-to-moderate stress hyperglycemia is protective because it provides a source of fuel for the immune system and brain at a time of stress [1]. However, many of the chronic endocrine responses result in persistent hyperglycemia and insulin resistance, which can be potentially deleterious in the long run [2]. In combination with inadequate systemic insulin levels and insulin resistance due to the increased secretion of counter-regulatory hormones, the negative manifestations inflicted by hyperglycemia lead to life-threatening conditions in these patients [3].

Incidences of stress-induced hyperglycemia, defined as plasma glucose levels exceeding 200 mg/mL in patients, have been documented for more than 100 years in patients experiencing severe trauma or injury [4]. Although hyperglycemia is believed to be an adaptive stress response, long-term stress-induced hyperglycemia is linked to poor clinical outcomes and increased risk of mortality [5]. The underlying causes of hyperglycemia during critical illness are attributed to the increased hepatic glucose production and impaired glucose consumption by peripheral tissues as well as insufficient pancreatic insulin production. In addition, the production and accumulation of counter regulatory hormones, such as glucagon, cortisol, catecholamines, and growth hormone, will increase lipolysis, protein breakdown, and impair glucose usage by peripheral tissues [4]. At the cellular level, increased blood glucose levels result in mitochondrial injury and endothelial dysfunction by generating reactive oxygen species and inhibiting nitric oxide production, respectively. Recently, it has been found that the endoplasmic reticulum (ER) stress response and its subsequent unfolded protein response are activated in various tissues under conditions related burn and severe trauma. ER stress has been identified as one of the central intracellular signaling pathways that link insulin resistance, hyperglycemia, and inflammation [6].

In this paper, we intend to review recent advances on the regulating effects of hyperglycemia and insulin on innate immunity, with a particular emphasis on severe burns. In addition, we will explore the history of insulin treatment on stress-induced hyperglycemia during critical illness and update the present understanding in regard to the ongoing moderate versus intensive insulin treatment debate. Although cytokine products and reactive oxygen species produced by innate immune cells may have profound effects on glucose disposal and utilization in the periphery as well as on insulin production by the pancreas, we only focus on the effects of hyperglycemia and insulin on innate immune cells.

## 2. Hyperglycemia and Innate Immune Cells 

### 2.1. Monocytes

Monocytes, macrophages, and dendritic cells are antigen-presenting cells that possess phagocytic capabilities that play a crucial role in maintaining immune homeostasis and mounting an immune response against infection. In severely burned and septic patients, monocyte phenotype and function are disrupted, resulting in a lowered expression of HLA-DR on circulating monocytes as early as 2-3 days after injury. This effect of decreased HLA-DR expression and cytokine production has shown to persist for 28 days in burn and septic patients [7].

The adverse effects of hyperglycemia on innate immunity manifest through the regulation of monocyte cytokine secretion. This notion is well supported by previous studies using THP-1 monocyte cell line and human peripheral blood monocytes, which when cultured under high glucose conditions caused elevated expression of MCP-1, TNF-*α*, IL-1*β* [8, 9], COX2 [10], IP-10 [11], and IL-6 [12]. A study using primary human monocytes shows that HG-induced TNF-*α* production is through the downregulation of CD33 [13].

Importantly, hyperglycemia-induced abnormal cytokine production in patients with severe sepsis exacerbates the clinical outcomes of these patients experiencing stress hyperglycemia [14]. Accumulating evidence indicates that IL-6 is involved in glucose metabolism and insulin action. The proinflammatory cytokine IL-6 is normally released upon infection; however, it induces insulin resistance during conditions of hyperglycemia [15]. Devaraj and Jialal found that increased secretion of IP-10 from monocytes cultured with high concentration glucose was via TLR2 and TLR4 pathway, since blockade of TLR2 and/or TLR4 inhibited IP-10 release [11]. Other studies further demonstrated that HG-induced TLR-2 and -4 expressions are via protein kinase C (PKC) activation and by the stimulation of NADPH oxidase [16].

However, other studies showed that cytokine secretion was inhibited in the presence of higher concentration of glucose or C-peptide using* in vitro* culture of U937 cell line or* ex vivo* culture of freshly isolated leukocytes from healthy volunteers. Inhibited cytokines include IL-6, IL-8, macrophage inflammatory protein- (MIP-) 1*α*, MIP-1*β* [17], TNF-*α*, and reactive oxygen species [18].

Furthermore, HG influences monocyte HLA-DR expression. Monocytes from healthy volunteers that were exposed for 24 hours to high concentrations of glucose (400 mg/dL) presented a decreased HLA-DR [19]. It indicates that HG may impair the antigen presenting activity of monocytes. Hyperglycemia has also shown to regulate other functions of monocytes, such as adhesion, migration, and transmigration. Nandy et al. observed that high concentration of glucose augmented monocyte adhesion to human umbilical vein endothelial cell monolayer and increased migration [20]. In contrast, another study showed that adhesion of monocytes to human aortic endothelial cells was diminished in the presence of 30 mM/L glucose and C-peptide [17]. The phagocytic ability of innate immune system has been found to be marginally enhanced by hyperglycemia [18].

Taken together, numerous studies have investigated the effects of hyperglycemia on monocyte functions including cytokine secretion and migration. As a result of the inconsistent findings, there is a lack of consensus on the relationship between monocytes and high glucose conditions and further investigation is required. The discrepancies may be resulted from the different model system and exposure time among investigators. We would like to emphasize that the effects of hyperglycemia on monocytes are not necessarily equivalent to the status of monocytes in severely injured patients, such as burned and septic patients. There are many other factors that contribute to monocyte phenotype in addition to hyperglycemia.

### 2.2. Macrophages

Macrophages are an essential component of the immune system, with three fundamental homeostatic activities: host defense, wound healing, and immune regulation. Due to the large number of macrophages in the tissues and its role as a major source of cytokines during injury, hyperactive macrophages are the leading contributors of systemic inflammatory response syndrome (SIRS). Interestingly, recent research has shown that macrophages are heavily involved not only in proinflammatory signaling cascades but are also pivotal in the phases of anti-inflammatory, wound healing, and sepsis in critical illness.

To examine the effects of high glucose on macrophage proliferation, Liu et al. cultured monocyte/macrophage cell line WEHI-3 and splenic macrophages in hyperglycemic media with various concentrations (5.6–30 mM) of glucose. They found that macrophage proliferation increased with the greater concentrations of glucose [21]. The enhanced macrophage proliferation may result from increased CSF-1 receptor (CSF-1R) under these conditions [22]. In addition, hyperlipidemia has combined effect with hyperglycemia to stimulate the proliferation of macrophages since hypermetabolisms including hyperglycemia and hyperlipidemia are very common in critical illness [23]. In addition, hyperglycemia also enhances the immunological responses, as is shown in that hyperglycemia augmented increased cytokine production and phagocytosis in response to LPS [24]. This effect may be associated with elevated TLR expression [16].

Extending the* in vitro* experiments using cell lines and isolated splenic macrophages, there has been an* ex vivo* study examining the effects of hyperglycemia on alveolar macrophage function [25]. In contrast to studies using cell lines, hyperglycemia significantly decreased the respiratory burst of alveolar macrophages and impaired proinflammatory cytokine secretion, such as TNF-*α* and IL-6. It also demonstrated a reduced response to multiple TLR ligands in alveolar macrophages. The impaired reactivity of alveolar macrophage to TLR ligands might result from HG-induced alteration of intracellular signaling and is unlikely due to the modulation of TLR expression itself [25]. Hyperglycemia also promotes the inflammatory response by activating the NF-*κ*B pathway. Using a rat model of hyperglycemia and burn injury, Kulp et al. investigated the effects of hyperglycemia on inflammatory responses in the liver. Streptozotocin-induced hyperglycemia in severely burned rats rapidly activated NF-*κ*B pathways in the liver and markedly increased liver acute-phase proteins and proinflammatory cytokines. On the contrary, long-term exposure to hyperglycemia leads to alternative activation of macrophage. F4/80(+) peritoneal exudate macrophages (PEMs) from mice with diabetes for 4 months displayed significantly reduced proinflammatory cytokines TNF-*α* and IL-6 production but enhanced nitric oxide (NO) secretion when treated with IFN-*γ* and LPS, while the activity of arginase in PEMs from diabetic mice was significantly higher than control mice when stimulating with IL-4 [26].

In summary, hyperglycemia has established itself as a regulator of macrophage proliferation and activity. The detrimental effects of hyperglycemia on thermal injury outcome may be mediated in part by augmenting macrophage inflammation via the activation of hepatic NF-*κ*B pathway [27].

### 2.3. Neutrophil

Neutrophils are typically the first leukocytes to be recruited to the inflammatory site and are capable of eliminating pathogens by multiple mechanisms. Following infection, the localization of neutrophils to the site of inflammation is crucial for the clearance of pathogens. When considering burn shock, the inflammatory response-related hyperactivation of neutrophil contributes to oxidative cell/tissue damage and potentially initiates organ-system dysfunction and failure. Severe burn and sepsis result in an inhibition of neutrophil function including migration [28] and neutrophil paralysis leads to increased rate of infectious complications in short-term hyperglycemic critically ill patients [29].

Hyperglycemia induces neutrophil dysfunction by modulating one of the neutrophil biochemical pathways, myeloperoxidase (MPO). MPO plays an important role in the killing function of neutrophils. Hyperglycemia also reduces neutrophil degranulation and exaggerates coagulation in healthy humans that accepted glucose infusion and injection with endotoxin [30]. Since glucose and glutamine play a key role in neutrophil function, changes in metabolism of neutrophils under the condition of hyperglycemia may play an important role in the impaired neutrophil function observed in diabetes [31]. Sustained decreases in neutrophil function associated with hyperglycemia are associated with the extent of hyperglycemia [32].

### 2.4. *γδ* T Cells

*γδ* T cells, a T-cell subset expressing *γδ* TCR, account for approximately 3–5% of all lymphoid cells found in the secondary lymphoid tissues and the blood. They are relatively abundant in the skin epithelia, intestine, uterus, and tongue where they can account for up to 50% of the total intraepithelial lymphocyte population.

Resident intraepithelial *γδ* T cells are responsible for maintaining epithelial integrity, regulating homeostasis and providing a first line of defense against pathogens and injury in mice and humans. Schwacha and collaborators found that *γδ* T cells play a role in neutrophil-mediated remote organ (i.e., lung, small intestine) injury early after burn injury by increasing chemokine levels in both the plasma and tissues [33]. Another study from the same group showed a 6-fold reduction in cellular infiltrate in burn wound and a marked decrease of levels of MCP-1, IL-6, and TNF-*α* in the wound in *γδ* T cells receptor-deficient mice [34]. More recently, a study showed that hyperglycemia negatively impacts homeostasis and functionality of skin *γδ* T cells. Hyperglycemia results in impaired skin *γδ* T cell proliferation, ultimately resulting in half the normal amount residing in the epidermis. These *γδ* T cells expressed decreased levels of NR4A1 and NR4A3, two orphan nuclear receptors that have been shown to sensitize muscle to insulin, suggesting their decreased insulin sensitivity. The dysfunctional *γδ* T cells can also result from the effects of chronic inflammatory mediators, such as TNF-*α*, in the local environment [35].

Overall, skin *γδ* T cells recognize epithelial damage and release cytokines and growth factors that facilitate wound repair. Their activities are compromised by hyperglycemia, rendering host defense mechanisms vulnerable to further injury and infection in patients with critical illness.

## 3. Insulin and Innate Immune Cells

Insulin exerts its effects on immune cells by binding to the insulin receptor (IR), that is, extensively expressed on immune cells, such as neutrophils and monocytes/macrophages. Upon insulin's binding to the IR, insulin rapidly increases tyrosine phosphorylation of its own receptor followed by the phosphorylation of the insulin receptor substrate proteins (IRS). The IRS is linked to the activation of two main signaling pathways: the phosphatidylinositol 3-kinase (PI3K)—AKT/protein kinase B (PKB) pathway and the Ras/mitogen-activated protein kinase (MAPK) pathway. It has been shown that mice deficient in insulin have an exaggerated cytokine response to peritoneal inflammation compared to controls, indicating that insulin treatment not only decreases glucose levels but also inhibits the inflammations [36]. Recent studies indicate that insulin levels vary in patients but that higher insulin levels may be associated with increased mortality, perhaps suggesting insulin resistance [37, 38]. In the following section, we will review insulin's effects on immune function and metabolism of innate immune cells (summarized in Table 1). For more detailed insulin signaling pathway, please refer to a decent review paper [39].

### 3.1. Monocytes

Insulin is considered to be a regulator of monocyte function, which includes chemotaxis, phagocytosis, and oxidative burst capacity. In a rabbit model of burn injury, researchers found that insulin improved the capacity for phagocytosis and oxidative burst within 3 days after burn, with no effect on chemotaxis [40]. A similar effect was observed in trauma patients receiving intensive insulin therapy, which showed enhanced monocyte phagocytosis [41].

Another role that insulin has is influencing the metabolism of monocyte. Insulin increased oxidized low-density lipoprotein phagocytosis of monocytes isolated from healthy obese participants [42]. The effects of insulin on the rates of glucose transport in monocytes were measured with the NBDG fluorescent d-glucose analog. Insulin caused an increase in the uptake of glucose and the expression of glucose transporter (GLUT) isoforms GLUT3 and GLUT4 in the plasma membrane [43].

Insulin also regulates chemokine and cytokine secretion of monocytes. The addition of insulin to human monocyte cell culture promotes IL-8/CXCL8 secretion. IL-8/CXCL8 is a potent chemoattractant for neutrophils and causes degranulation of neutrophil-specific granules and azurophilic granules. These results suggest that insulin may regulate the recruitment and activity of neutrophils by inducing IL-8/CXCL8 secretion from monocytes [44]. THP-1 monocytes incubated with insulin and palmitate together produced more IL-6 and TNF-*α*, compared to monocytes incubated with palmitate alone. However, incubation of monocytes with insulin alone did not affect the production of IL-6 or TNF-*α* [45]. Hyperinsulinemia also influences monocytic HLA-DR expression. Monocytes from healthy volunteers were treated with insulin (concentration from 10 *μ*U to 200 *μ*U) for 24 hours* in vitro* and monocytic HLA-DR was significantly decreased in a dose-dependent manner [19].

Lastly, insulin also regulates other activities of monocytes including superoxide production and the expression of tissue factor (TF) and MMP-9. It stimulates superoxide (O_2_^−^) production in monocytes and macrophages [46], which is dependent on NADPH oxidases. NADPH oxidase plays a pivotal role in insulin-induced activation of monocytes [46]. Insulin may influence the hypercoagulability in patients by inhibiting tissue factor expression in monocyte, the principal initiator of the extrinsic coagulation pathway [47].

### 3.2. Macrophages

Generally speaking, insulin attenuates the immune response of macrophages. It inhibits TNF-*α* and IL-8 secretion by macrophage in response to LPS. This effect is via the release of activin A and the signaling by cytoplasmic SH2-containing inositol 5′-phosphatase (SHIP) [48]. It also modulates tissue inflammation by reducing macrophage accumulation in visceral adipose tissue in mice [49]. Another study also showed that insulin inhibits cytokine secretion (TNF-*α* and IL-1*α*) by macrophage and improves its survival [50]. Pretreatment of cells with specific covalent inhibitor of phosphoinositide 3-kinases significantly inhibited insulin-mediated cell survival and BclXL expression. In addition, the enhancing effect of insulin on BclXL expression is dose-dependent [51]. Furthermore, macrophages from mice with streptozotocin- (STZ-) induced diabetes display a dysfunctional phenotype, reduced CD86 expression, and proinflammatory cytokines such as TNF-*α* and IL-6 production but enhanced nitric oxide (NO) secretion [26]. These functional changes of macrophages could be efficiently reversed by insulin treatment and this effect is dependent on the activities of AKT and ERK [26].

Insulin also regulates macrophage metabolism. Recent study shows that insulin promotes human macrophage foam cell formation by increasing type II scavenger receptor CD36 and decreasing the expression of the ATP-binding cassette transporter ABCA1. As a result, it leads to 2-3-folds more cholesterol accumulation within a short period by increasing oxidized LDL uptake and decreasing cholesterol efflux to apolipoprotein A1 (apoA1) [52]. Insulin also promotes foam cell formation by accelerating endocytic uptake of advanced glycation end products (AGE) proteins [53]. In addition, insulin specifically promotes the protein degradation of LRP1 and therefore decreases LRP1 expression on macrophages. The decreased expression of LRP1 impairs the cellular internalization of alpha-2-macroglobulin, which may modulate cytokine secretion by macrophage [54].

### 3.3. Neutrophils

*In vitro* study indicated that insulin regulated isolated neutrophil cytokine secretion. Activin A, a transforming growth factor-*β* family cytokine, plays a crucial role in regulating the onset and severity of many inflammatory conditions. Bone marrow-derived neutrophil precursors contained 7-fold higher concentrations of activin A than bone marrow mononuclear cells. These isolated neutrophils could release activin A in response to TNF-*α*. However, production of activin A would be blocked upon pretreatment with insulin [55].

The* in vivo* effects of insulin on neutrophils were conducted in healthy subjects under strict euglycemia and physiological insulinemia. They found that insulin increased the total number of neutrophils and the number of these expressing CD11b, CD15, CD62L, and CD89, whereas the density of these molecules was downregulated. In addition, insulin increased PMN function including chemotaxis, phagocytosis, and bactericidal capacities [56]. Interestingly, although insulin stimulated phagocytosis and bactericidal activity in young-adult subjects, these effects were compromised in the elderly subjects [57]. Studies of patients who underwent major surgery showed that insulin treatment not only significantly decreased the level of blood glucose, but it also increased the number of neutrophils in the circulation as well as their ability to ingest foreign particles [58].

## 4. Intensive versus Moderate Insulin Treatment

Stress hyperglycemia leads to an increased incidence of infection and higher morbidity and mortality in severely traumatic patients [59]. To manage hyperglycemia in patients with severe trauma and illness, van den Berghe and colleagues conducted the first clinical study of IIT over 10 years ago [2]. The study showed that maintaining blood glucose at or below 110 mg/dL by IIT reduces morbidity and mortality among critically ill patients in the surgical intensive care unit. With a total of 1548 patients enrolled, IIT reduced mortality rate from 8% with conventional treatment to 4.6% [2]. Intensive insulin protocols in thermally injured patients have shown improved wound healing by 6.9% in the early stages in comparison to burn controls [60]. Severely burned pediatric patients had reduced urinary tract infections and sepsis in the IIT group with a positive association with survival [61]. Early control of hyperglycemia is essential since a lack of early glycemic control (mean daily blood glucose < 150 mg/dL in at least two consecutive days by postburn day 3) was associated with higher mortality [62].

Despite the vast benefits of tight glycemic control with IIT, it is accompanied by a mandate for critically monitoring and awareness of sudden fluctuations in blood glucose. There was no overall impact on hospital or ICU length of stay in severely burned paediatric patients who received IIT [60]. In addition, there was a significant concern regarding the use of IIT in managing elevated blood glucose in traumatic brain injury patients. IIT did not result in reduced cerebral metabolic rate, but it did increase markers of neural metabolic distress and showed no improvement in mortality [63]. More recently, IIT in both critically ill neurological and stroke patients showed more episodes of hypoglycemia and little to worsening effect of patient outcomes compared to nonaggressive approaches [64].

There has been an ongoing debate regarding the effectiveness of IIT versus modest insulin treatment [65]. In fact, hyperglycemia can be safely avoided using a moderate glycemic control protocol without inducing hypoglycemia [66]. Using a retrospective approach, Kutcher and colleagues concluded that the two treatments had no significant impact on multiorgan failure and mortality. However, the moderate regime had scarce hyperglycemia episodes, low glucose variability, and intermediate blood glucose ranges of hypoglycemia [66].

Along with the numerous investigations of insulin therapy to manage stress hyperglycemia, there are numerous other treatment options that have been studied. The use of metformin in treating hyperglycemia decreases endogenous glucose production and increases glucose clearance and oxidation [67]. The use of other agents in combination with insulin, such as glucagon-like peptide-1 (GLP-1) analogue exenatide, has shown to reduce the amount of insulin required to achieve euglycemia [4]. Lastly, the lipolysis agonist Fenofibrate treatment can reduce insulin resistance [68] and, when used in combination with insulin, reduces hypoglycemic episodes with clear improvements in skeletal muscle insulin signaling, glucose oxidation, and mitochondrial function [69].

There is compelling evidence for the multifaceted effect of hyperglycemia treatment and the respective outcomes in critically injured and burn patients. Patients will benefit most from the use of moderate insulin treatment regimens with rigorous attention being given in the first few days of injury to obtain blood glucose levels within the well-established target ranges (summarized in Table 2). Future prospective randomized trials need to place emphasis on the frequency of hypoglycemic and hyperglycemic episodes and the extreme changes in glucose variability to determine the detrimental impact on survival.

## 5. Summary 

**Figure 1:**
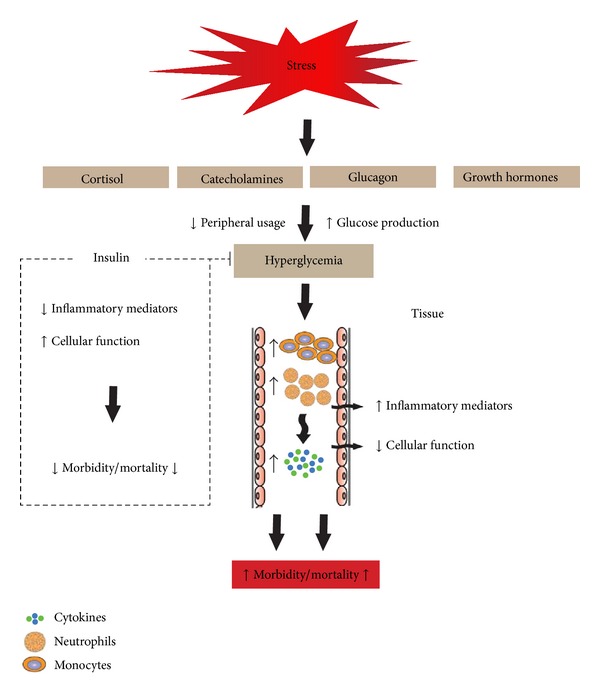
Schematic summary of hyperglycemia and insulin treatment regulation of innate immune cells. Overwhelming stress resulted from critical illness, such as severe burn, major surgery, or sepsis stimulates the release of cortisol, catecholamines, glucagon, and growth hormone, which increase hepatic glucose production and impair glucose consumption by peripheral tissues. Long-term stress-induced hyperglycemia induces hyperproinflammatory responses and depressed cell functions, which is linked to increased risk of mortality and morbidity. Insulin plays a different role in regulating innate immune cells including monocytes, macrophages, and neutrophils. It generally improves their cellular activities and attenuates their inflammatory responses.

Stresses-induced insulin resistance and hyperglycemia represent adverse sequelae resulting from trauma and critical care injuries. Long-term exposure to stress-induced hyperglycemia is linked to an increased incidence of infections and sepsis, multiorgan failure, and mortality. Both hyperglycemia and insulin have profound effects on the function of innate immune cells. Hyperglycemia tends to favor the proinflammatory immune response. Insulin has not only anabolic, but also anti-inflammatory and immune regulatory properties as shown in Figure 1. The interaction or integration of hyperglycemia and insulin on the innate immune cells should be investigated desirably in the future. Animal models of stress-induced hyperglycemia are challenging. A recent study indicates that genomic responses in mouse models poorly mimic human inflammatory diseases including burn and sepsis. In this regard, it is the priority for translational research to take advantage of the clinical samples rather than relying on mouse models to study human inflammatory diseases [70]. Furthermore, to increase the study depth we suggest that future studies should examine the effects of the combined effects of hyperglycemia and hyperlipidemia on innate immune cells since hyperlipidemia is also the hallmark of critical illness.

Glycemic control established early (2-3 days after trauma or burn) in critically ill hyperglycemia patients leads to reduce mortality. Modest glycemic control has much fewer hyperglycemic episodes and a lower frequency of hypoglycemia. The combination of insulin treatment with other agents will reduce the insulin dose and hypoglycemic episodes. A better understanding of the roles played by hyperglycemia and insulin in the regulation of innate immunity will guarantee a more rational and an effective insulin treatment for these patients.

## Figures and Tables

**Table 1:** Effects of hyperglycemia and insulin on innate immune cells.

	Hyperglycemia	Insulin
Monocyte	(1) Generally enhances cytokine production [7–10, 13–15] (2) Regulates adhesion, migration, and transmigration [13, 14, 17]	(1) Enhances pathogen clearance [34, 35] (2) Promotes IL-8/CXCL8 secretion [38] (3) Increases superoxide production [40] (4) Promotes TNF-*α* and IL-6 secretion in the presence of palmitate [37] (5) Regulates monocyte metabolism by increasing the phagocytosis of oxidized low-density lipoprotein [36, 37]

Macrophage	(1) Promotes proliferation [18, 19] (2) Enhances cytokine production and phagocytosis in response to LPS *in vitro* [16, 20] (3) Impairs proinflammatory cytokine secretion, such as TNF-*α* and IL-6 *ex vivo* [21]	(1) Inhibits TNF-*α*, IL-1, and IL-8 secretion [42, 44] (2) Reduces macrophage accumulation in tissue [43] (3) Promotes human macrophage foam cell formation [47, 48]

Neutrophil	(1) Inhibits neutrophil function such as degranulation [25–27] (2) Downregulates production of myeloperoxidase (MPO) [25]	(1) Increases the total number of PMN and their surface expression of CD11b, CD115, CD62L, and CD89 [50] (2) Increases PMN function including chemotaxis, phagocytosis, and bactericidal capacities [50, 51]

*γδ* T cells	(1) Impairs skin T cell proliferation [30] (2) Inhibits neutrophil tissue infiltration [28, 29]	N/A

**Table 2:** Comparison of intensive and moderate insulin treatment.

	Intensive	Moderate
Target	Blood glucose: 110 mg/dL [1]	Blood glucose: 120–150 mg/dL [33, 59]

Advantages	(1) Improves wound healing in burned patients [54] (2) Reduces urinary tract infections and sepsis in burned pediatric patients [55] (3) Reduces morbidity and mortality [1]	(1) Does not induce hypoglycemia [60] (2) Scarce hyperglycemia episodes [60] (3) Low glucose variability [60]

Disadvantages	(1) Requires continual and critical monitoring [54] (2) No overall impact on hospital or ICU length of stay [54] (3) More episodes of hypoglycemia [58]	No significant impact on mortality and multiorgan failure [60]
